# Biochemical Insights into a Novel Family 2 Glycoside Hydrolase with Both β-1,3-Galactosidase and β-1,4-Galactosidase Activity from the Arctic

**DOI:** 10.3390/md21100521

**Published:** 2023-09-29

**Authors:** Dianyi Li, Zheng Wang, Yong Yu, Huirong Li, Wei Luo, Bo Chen, Guoqing Niu, Haitao Ding

**Affiliations:** 1 College of Agronomy and Biotechnology, Southwest University, Chongqing 400715, China; lidianyi2020@163.com; 2 Antarctic Great Wall Ecology National Observation and Research Station, Polar Research Institute of China, Ministry of Natural Resources, Shanghai 200136, China; wangzheng01@pric.org.cn (Z.W.); yuyong@pric.org.cn (Y.Y.); lihuirong@pric.org.cn (H.L.); luowei@pric.org.cn (W.L.); chenbo@pric.org.cn (B.C.); 3 Key Laboratory for Polar Science, Polar Research Institute of China, Ministry of Natural Resources, Shanghai 200136, China; 4 School of Oceanography, Shanghai Jiao Tong University, Shanghai 200030, China

**Keywords:** β-galactosidase, *Marinomonas*, glycoside hydrolase, Arctic

## Abstract

A novel GH2 (glycoside hydrolase family 2) β-galactosidase from *Marinomonas* sp. BSi20584 was successfully expressed in *E. coli* with a stable soluble form. The recombinant enzyme (rMaBGA) was purified to electrophoretic homogeneity and characterized extensively. The specific activity of purified rMaBGA was determined as 96.827 U mg^−1^ at 30 °C using ONPG (*o*-nitrophenyl-β-D-galactopyranoside) as a substrate. The optimum pH and temperature of rMaBGA was measured as 7.0 and 50 °C, respectively. The activity of rMaBGA was significantly enhanced by some divalent cations including Zn^2+^, Mg^2+^ and Ni^2+^, but inhibited by EDTA, suggesting that some divalent cations might play important roles in the catalytic process of rMaBGA. Although the enzyme was derived from a cold-adapted strain, it still showed considerable stability against various physical and chemical elements. Moreover, rMaBGA exhibited activity both toward Galβ-(1,3)-GlcNAc and Galβ-(1,4)-GlcNAc, which is a relatively rare occurrence in GH2 β-galactosidase. The results showed that two domains in the C-terminal region might be contributed to the β-1,3-galactosidase activity of rMaBGA. On account of its fine features, this enzyme is a promising candidate for the industrial application of β-galactosidase.

## 1. Introduction

β-Galactosidase (EC 3.2.1.23, BGA), also commonly known as lactase, is an important glycoside hydrolase (GH) that is widely distributed in plants, animals and microorganisms [1]. The enzyme can catalyze molecules containing galactose group to break its terminal β-glycosidic bond and to transfer β-galactosyl group to corresponding receptors depending on the reaction system [2,3]. When a water molecule acts as a receptor, β-galactose is generated as one of the products; however, when monosaccharides, disaccharides or oligosaccharides are employed as receptors, galacto-oligosaccharides are produced rather than β-galactose. On account of its inherent characteristics, β-galactosidase has shown great application potential in pharmaceutical, biological and food industries [1,3,4], such as for the hydrolysis of lactose in dairy products for the treatment of lactose-intolerant people and production of galacto-oligosaccharides, one of the important human prebiotics [5,6]. In addition, β-galactosidase has also been used to hydrolyze whey, which is a by-product of cheese that causes water contamination, and can be hydrolyzed by β-galactosidase and converted into more valuable products such as ethanol and sweet syrup [7,8]. On a commercial and industrial scale, the most commonly used β-galactosidases are those derived from microorganisms, mainly *Aspergillus* and *Kluyveromyces* [1,3].

Based on the amino acid sequence, β-galactosidases can be classified into different glycoside hydrolase families including GH1, GH2, GH35, GH39, GH42, GH59, GH147, GH165 and GH173 (http://www.cazy.org, accessed on 26 April 2023), the main ones being GH1, GH2, GH35, and GH42 [3,9]. The largest variety of β-galactosidases are found in the GH2 family. Most of the enzymes found in the GH35 family are β-galactosidases, and all plant-derived β-galactosidases belong to this family [9,10]. Most GH42 family β-galactosidases are identified from microorganisms isolated from extreme environments, and the GH42 family also showed a broader substrate specificity, being able to hydrolyze substrates with different glycosidic bond linkages [11,12]. All β-galactosidases share the same (α/β)_8_ barrel structure and belong to the superfamily Clan-A [13,14].

As galactose in nature is usually linked to other molecules through β-1,3, β-1,4, or β-1,6 glycosidic bonds [15], β-galactosidases can also be classified as β-1,3-galactosidase, β-1,4-galactosidase and β-1,6-galactosidase based on their recognition of glycosidic bonds. As a rule of thumb, β-1,4-galactosidases are the most commonly found in nature, and only a few enzymes capable of specifically hydrolyzing β-1,3 or β-1,6-linkage of the nonreducing end galactosyl residue have been identified [12,16,17,18]. Furthermore, most β-1,3-galactosidases and β-1,6-galactosidases were found in the GH35 and GH42 families [19,20,21,22,23,24,25,26]. Generally, the extreme environments of the polar regions, such as extremely cold and dry, strong radiation and winds, shape extreme microorganisms, of which the enzymes evolved specific structures and functions to adapt the special environment [27,28]. Therefore, polar microorganisms are an important source of enzyme resources for people who want to obtain enzymes with specific functions. In this study, a novel family 2 glycoside hydrolase with both β-1,3-galactosidase and β-1,4-galactosidase activity was discovered from a *Marinomonas* strain isolated from the Arctic. The enzyme was successfully heterologously expressed in *Escherichia coli* and characterized extensively.

## 2. Results and Discussion

### 2.1. Sequence Analysis

The gene *mabga* encodes a peptide consisting of 869 amino acids, with a calculated molecular weight of 96.68 kDa and a theoretical pI of 5.48. The BlastP search against the NCBI database showed that MaBGA had the highest identity of 91.30% with a putative glycoside hydrolase family 2 TIM barrel-domain containing protein from *Marinomonas* sp. UCMA3892 (WP_169458495.1). However, MaBGA had low identities of about 30% with its well-characterized homologous proteins, suggesting that MaBGA might be a novel family 2 glycoside hydrolase. Sequence analysis of MaBGA via Pfam (http://pfam-legacy.xfam.org/, accessed on 20 September 2022) showed that this enzyme was composed of five domains, including three GH2 glycosyl hydrolases domains from position 35 to 656, a domain with unknown function from position 672 to 731 and a malectin domain at the C-terminus.

**Figure 1:**
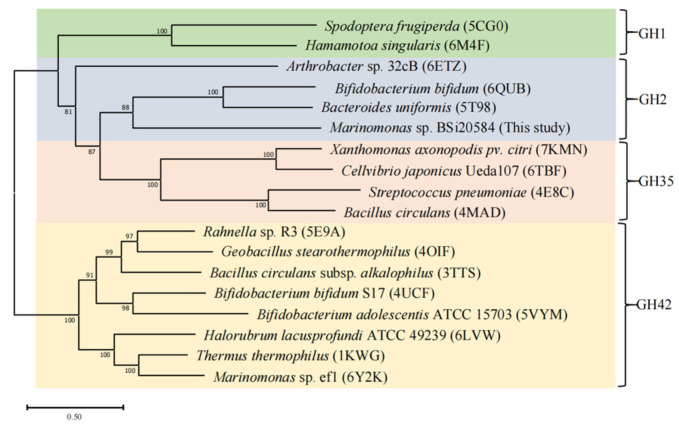
Unrooted phylogenetic tree of β-galactosidases belonging to the GH1, GH2, GH35 and GH42 families. The PDB accession number was provided following the species name.

**Figure 2:**
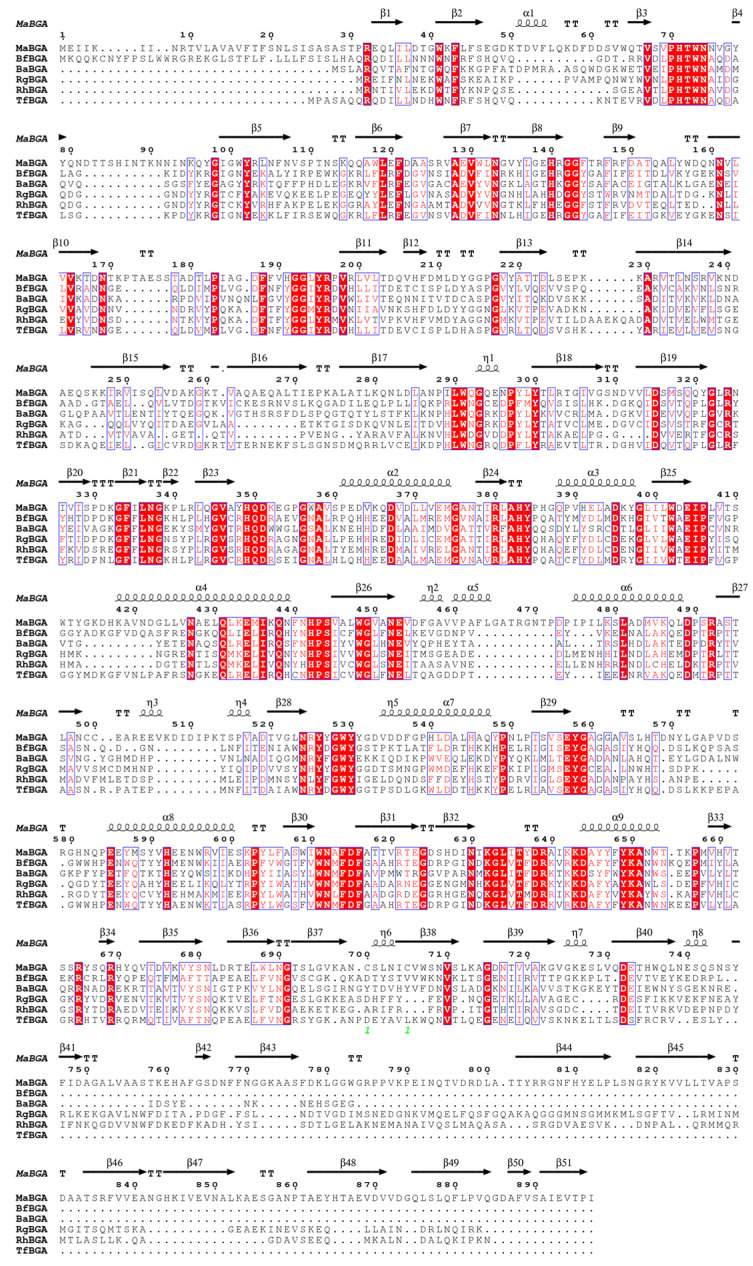
Multiple sequence alignment of structure-solved GH2 family β-galactosidases. Helices, β-sheets and turns are represented by squiggles, arrows and TT letters, respectively. Identical residues are shaded red and conserved substitutions are surround with blue lines. Abbreviations: β-galactosidase from *Marinomonas* sp. BSi20584 (this study), *Bacteroides fragilis* NCTC 9343 (3CMG), *Bacteroides fragilis* NCTC 9343 (3FN9), *Ruminococcus gnavus* (6MVG), *Roseburia hominis* (7KGZ) and *Tannerella forsythia* (8DHE) are represented as MaBGA, BfBGA, BaBGA, RgBGA, RhBGA and TfBGA, respectively.

Phylogenetic analysis of BGAs belonging to GH1, GH2, GH35 and GH42 also implied that MaBGA fell into the GH2 family branch (Figure 1). Additionally, multiple alignments of amino acid sequence of characterized GH2 family β-galactosidases displayed that MaBGA shared conserved domains with its GH2 counterparts (Figure 2).

### 2.2. Expression and Purification

Generally, signal peptide is located at the N-terminus of proteins, which plays an important role in the transfer of cell-surface proteins, however, the presence of signal peptide might affect the correct folding of proteins, resulting in a decrease in protein solubility. Therefore, removal of the signal peptide is often an effective approach to enhance soluble expression of heterologous proteins. In this study, the first 28 amino acids of MaBGA were predicted as signal peptides using the SignalP 5.0 online server. Based on the prediction, the recombinant plasmid pET-28a-MaBGA with the signal peptide removed was constructed. The results showed that the total activity and specific activity of the signal-peptide-free MaBGA increased by more than four times compared to MaBGA which retained a signal peptide, indicating that the removal of a signal peptide of MaBGA could enhance the solubility of the enzyme significantly. Subsequently, the signal peptide removed MaBGA was used for the next study.

**Figure 3:**
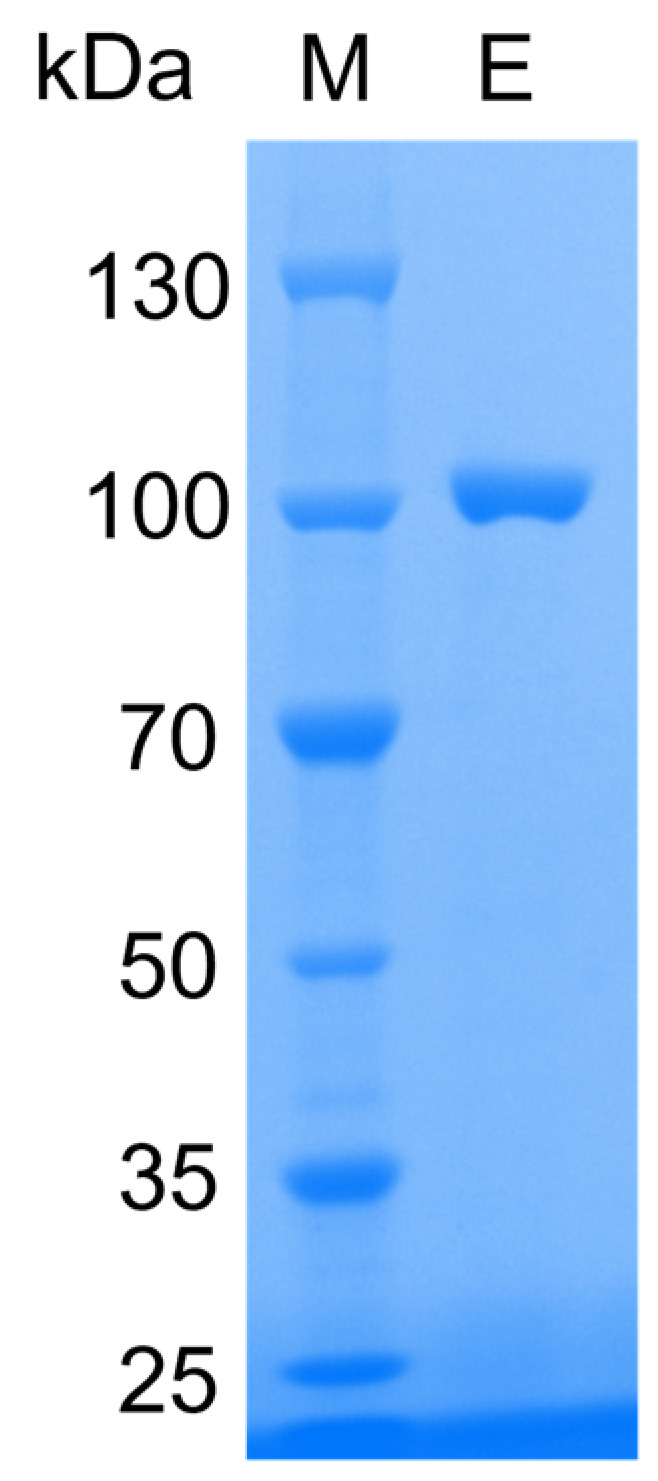
SDS-PAGE (sodium dodecyl sulfate polyacrylamide gel electrophoresis) analysis of the enzyme. Lane M: protein molecular weight marker; Lane E: purified rMaBGA.

The recombinant enzyme was heterologously expressed and purified via one-step immobilized metal affinity chromatography (IMAC). Electrophoretic analysis showed a homogeneous band corresponding to 100 kDa as predicted (Figure 3), indicating that rMaBGA had been purified to electrophoretic purity. The specific activity of rMaBGA was 96.827 U/mg at 30 °C using ONPG as the substrate.

### 2.3. Effects of Temperature on the Activity and Stability of the Enzyme

**Figure 4:**
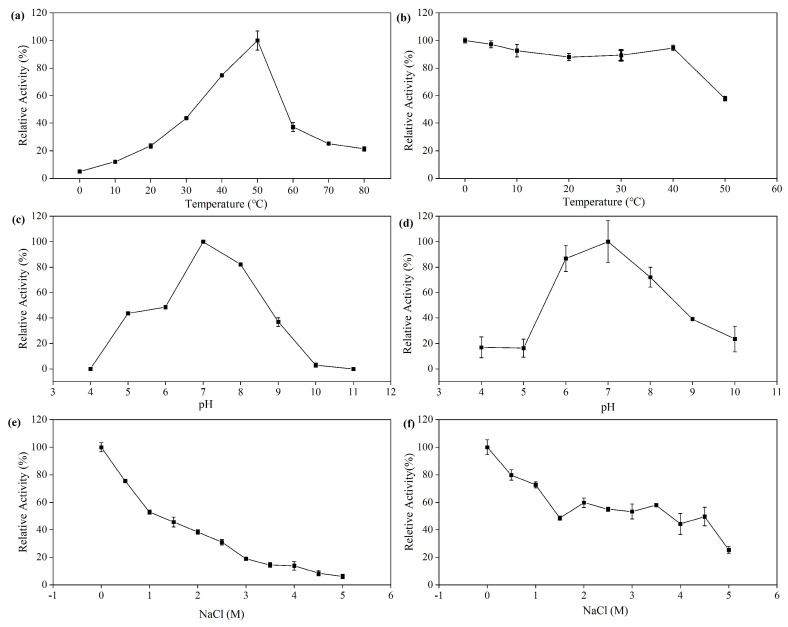
Effects of temperature, pH and NaCl on the activity and stability of rMaBGA. (**a**) Effect of temperature on the activity of rMaBGA; (**b**) effect of temperature on the stability of rMaBGA after incubation for 1 h; (**c**) effect of pH on the activity of rMaBGA; (**d**) effect of pH on the stability of rMaBGA after incubation for 1 h; (**e**) effect of NaCl on the activity of rMaBGA; (**f**) effect of NaCl on the stability of rMaBGA after incubation for 1 h. Data represented as mean ± standard deviation of triplicate measurements.

The recombinant enzyme displayed activity in a broad temperature range from 0 to 80 °C with the maximum activity determined at 50 °C; however, the activity of the enzyme decreased fiercely at temperatures above 50 °C (Figure 4a). Consistent with its activity profile, the enzyme was stable only at the temperature below 40 °C after incubation for 1 h, and the residual activity of rMaBGA dropped to approximately 60% of its initial activity after incubation at 50 °C for 1 h (Figure 4b). In addition, the enzyme exhibited high thermal stability at low temperature, which still retained 100% of its initial activity after incubation at 0–10 °C for 7 days. Although MaBGA is derived from a cold-adapted strain, it behaves like a mesophilic enzyme. Previous studies have found that many β-galactosidases from cold-adapted strains displayed high thermal stability and optimal temperatures; for example, the optimal temperatures of β-galactosidases from cold-adapted strains *Alteromonas* sp. ANT48 [29] and *Marinomonas* ef1 [30] are 50 °C and 60 °C, respectively.

### 2.4. Effects of pH on the Activity and Stability of the Enzyme

As shown in Figure 4c, rMaBGA displayed its maximum activity at pH 7.0, and the activity decreased sharply no matter whether the pH value was below or above 7.0, indicating a weak resistance to acidic and alkaline pH of the enzyme. A similar pattern was also observed in the pH stability profile of rMaBGA, which was only stable at neutral pH after incubating for 1 h in buffers with different pH values (Figure 4d).

### 2.5. Effects of NaCl on the Activity and Stability of the Enzyme

The activity of rMaBGA was highest when no extra NaCl was contained in the reaction system, as shown in Figure 4e; it gradually decreased with the increase in NaCl concentration. In addition, the recombinant enzyme nearly lost its total activity when 5 M of NaCl was added. Likewise, NaCl also showed a negative effect on the stability of rMaBGA, which was unstable after incubating for 1 h in buffers with different concentrations of NaCl added (Figure 4f).

### 2.6. Effects of Metal Ions and Chemicals on the Activity of the Enzyme

The activity of rMaBGA was slightly inhibited by monovalent cations including K^+^ and Na^+^, and some divalent cations such as Fe^2+^ and Ca^2+^. On the other hand, some divalent cations, Zn^2+^, Mg^2+^ and Ni^2+^, could improve the activity of the enzyme by up to 42% (Table 1). In addition, glycerol and oxidized L-glutathione also promoted the activity of the recombinant enzyme, while EDTA·2Na and SDS could significantly inhibit that of rMaBGA. Taking the effect of EDTA in conjunction with that of some divalent cations on the activity of rMaBGA into consideration, it is proposed that some divalent cations are vital to the catalytic process of rMaBGA.

### 2.7. Effects of Organic Solvents on the Stability of the Enzyme

**Figure 5:**
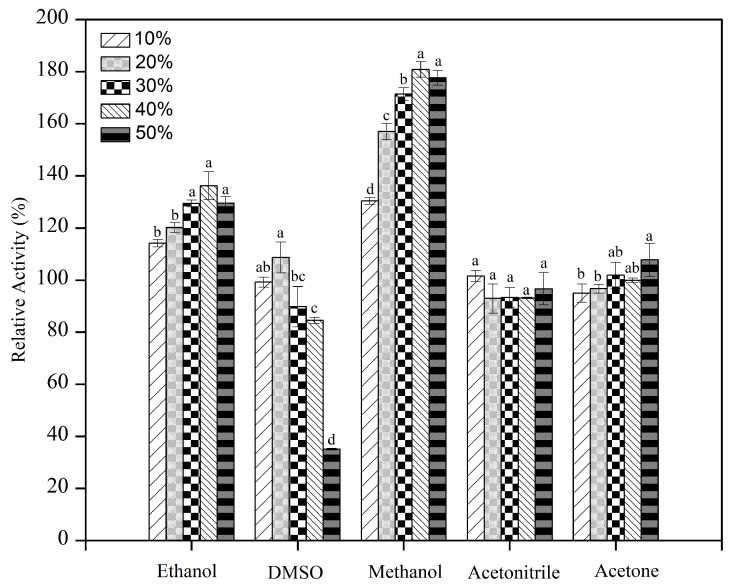
Effects of organic solvents on the stability of rMaBGA. Data represented as mean ± standard deviation of triplicate measurements. Different letters indicate statistically significant differences (*p* ≤ 0.05).

The results showed that the recombinant enzyme displayed strong resistance against water-soluble organic solvents, especially for methanol and ethanol, which even showed promoting effects with regard to the activity of rMaBGA after treatment of the enzyme by these organic solvents (Figure 5). The residual activity of rMaBGA increased with the increase in the methanol and ethanol concentration. As for acetonitrile and acetone, the increase in the concentration of organic reagents had no significant effect on the stability of the enzyme. DMSO had the greatest effect on the stability of rMaBGA, and when the volume fraction of DMSO reached 50%, only about 40% of rMaBGA activity remained. The one-way ANOVA showed that there was a highly significant difference between different concentrations of ethanol, DMSO, and methanol (*p* ≤ 0.001), while there was no significant difference between different concentrations of acetone and acetonitrile (*p* > 0.05).

### 2.8. Substrate Specificity of the Enzyme

The substrate specificity of rMaBGA was assayed using different sugars as the substrate. As shown in Table 2, the recombinant enzyme has the highest hydrolytic activity for its natural substrate β-lactose and weak activity towards other sugars, including trehalose, maltose, cellobiose, arabinogalactan and pectic galactan, indicating a strict substrate specificity of the enzyme.

### 2.9. Steady-State Kinetics

The steady-state kinetic constants of rMaBGA were determined using a nonlinear fitting plot (Table 3). When we compared the kinetic parameters among these substrates, ONPG had the lowest value of *K_m_* and the greatest value of *k_cat_*, indicating that the enzyme has the highest specificity toward ONPG, even higher than its natural substrate β-lactose. Furthermore, the enzyme also showed activity toward Galβ-(1,3)-GlcNAc, as well as Galβ-(1,4)-GlcNAc.

Generally, the great majority of β-1,3-galactosidases were found in the GH35 and GH42 families [19,20,21], few studies reported that β-galactosidase ascribed to the GH2 family showed weak activity toward Galβ-(1,3)-GlcNAc [11]. Compared to other common GH2 β-galactosidases such as LacZ from *Escherichia coli*, MaBGA has two different domains at the C-terminal region, as mentioned above. Previous studies have shown that the diversity of GH2 enzyme evolution is driven by the addition of different non-catalytic structural domains to the C-terminal region [31]. Therefore, a truncated mutant without these two domains carried was constructed and purified to electrophoretic purity, to investigate their roles in the catalytic activity towards Galβ-(1,3)-GlcNAc. The results showed that the truncation of these two domains did not affect the activity towards Galβ-(1,4)-GlcNAc but resulted in a loss of catalytic ability towards Galβ-(1,3)-GlcNAc, suggesting that the truncation region of MaBGA might be involved in the recognition of β-1,3-galactosidic bonds.

### 2.10. Thermal Denaturation Kinetics

The thermal denaturation kinetics constants of rMaBGA were measured using incubating enzymes at different temperatures (Table 4). The enzymes exhibited stable thermal dynamic features at temperatures below 40 °C. The half-life was determined as 59.94 h at 40 °C, which was approximately 16 times greater than that at 50 °C. The values of ΔH*, ΔG* and ΔS* of rMaBGA decreased with the increase in temperature, suggesting that the conformation of the enzyme was changed by heat treatment.

## 3. Materials and Methods

### 3.1. Strains, Culture Conditions and Reagents

*Marinomonas* strain BSi20584 isolated from sea ice in the Arctic Ocean was used as the source of β-galactosidase in this study. *Escherichia coli* strains DH5α and BL21 (DE3) cultivated in Luria Broth medium at 37 °C, were used for gene cloning and expression, respectively. Plasmid pET28a (+) was employed to construct the recombinant vector. Substrates Galβ-(1,3)-GlcNAc and Galβ-(1,4)-GlcNAc were obtained from TRC (Toronto, ON, Canada). Ni-NTA Agarose was purchased from Qiagen (Dusseldorf, Germany). DNA marker and Taq polymerase were obtained from Takara (Dalian, China). All other chemicals used in this study were of analytical grade.

### 3.2. Gene Cloning and Sequence Analysis

The nucleotide sequence, derived from *Marinomonas* sp. BSi20584 (NCBI: GCA_002792415.1), encoding for a putative β-galactosidase (MaBGA) was optimized for better soluble expression in *Escherichia coli*, with the CAI (Codon Adaptation Index) upgrading to 0.9 and the average GC content adjusting to 51.91%. The optimized gene sequence was synthesized with the cleavage sites of *Nde*I and *Xho*I carried and ligated with the pre-digested vector pET-28a (+), to construct recombinant plasmid pET-28a-MaBGA-FL. For further optimization of the soluble expression of the enzyme, the signal peptide of MaBGA was removed based on the prediction of SignalP 5.0 online server (https://services.healthtech.dtu.dk/services/SignalP-5.0/, accessed on 20 December 2022). The gene encoding for signal-peptide-removed MaBGA was amplified using recombinant plasmid pET-28a-MaBGA-FL as a template with the forward primer 5′-GGAATTCCATATGTCTACCCCGCGTGAACAGCT-3′ and the reverse primer 5′-CCGCTCGAGTTAGGTGGTGGTGCTCGAGG-3′, carrying the cleavage sites of *Nde*I and *Xho*I (underlined), respectively. The amplified fragment was recovered by gel and ligated with vector pET-28a (+) to obtain the plasmid pET-28a-MaBGA with the signal peptide removed. To investigate the potential function of the C-terminal domain of MaBGA, the recombinant plasmids pET-28a-rMaBGA-Trun with the first 1-643 amino acids retained were constructed with the same method using forward primer 5′-GGAATTCCATATGTCTACCCCGCGTGAACAGCT-3′ and reverse primer 5′-CCGCTCGAGGGTGTTGATGTCGTGGGAAT-3′. Further, we focused on rMaBGA-Trun (retaining the first 1-643 amino acids) to explore the catalytic function of the C-terminus using the protein truncation test. The recombinant plasmid was transformed into competent cells of *E. coli* DH5α for sequencing and then transformed into competent cells of *E. coli* BL21 (DE3) for expression.

A homologous search was performed using the BLAST server (https://blast.ncbi.nlm.nih.gov/Blast.cgi, accessed on 22 August 2023). Alignment of protein sequences was implemented using Clustal Omega software [32] and rendered by ESPript 3.0 [33]. The phylogenetic tree was constructed in MEGA 11 using the neighbor-joining method [34], with a bootstrap test of 1000 replicates.

### 3.3. Gene Expression and Protein Purification

Recombinant cells were cultured to the value of OD_600_ in the range from 0.6 to 0.8 in LB medium at 37 °C with a rotatory speed of 150 rpm, and IPTG was added to the medium at a final concentration of 0.1 mM to induce gene expression for another 16 h at 20 °C. After induction, cells were collected via centrifugation and washed twice with PBS buffer (137 mM NaCl, 2.7 mM KCl, 10 mM Na_2_HPO_4_, 1.8 mM KH_2_PO_4_, pH 7.4). The pellet was resuspended in PBS buffer and disrupted with ultrasonication. Cell debris was removed via centrifugation at 7600 rpm for 30 min. The supernatant was filtered through a syringe filter with 0.45 μm pore size and then loaded onto a column filled with pre-equilibrated Ni-NTA resin. The resin was washed with wash buffer (50 mM NaH_2_PO_4_, 300 mM NaCl, 40 mM imidazole, pH 8.0) and eluted with elution buffer (50 mM NaH_2_PO_4_, 300 mM NaCl, 250 mM imidazole, pH 8.0). The eluted enzyme was desalted and concentrated via ultrafiltration and stored at −80 °C. The protein concentration was measured according to the Bradford method using BSA as a standard. All purification steps were carried out at 4 °C.

### 3.4. Electrophoretic Analysis

The purified protein was analyzed using SDS-PAGE on a gradient gel (4–15% of Tris-Gly) and stained with Coomassie Brilliant Blue R-250. The molecular weight of the recombinant enzyme was estimated by comparing its electrophoretic mobility with a pre-stained protein marker obtained from Sangon (Shanghai, China).

### 3.5. β-Galactosidase Activity Assay

The activity was measured by monitoring the absorbance of ONP (o-nitrophenyl) at 420 nm in 10 mM PBS buffer with 10 mM ONPG as substrate. One unit of enzyme activity was defined as the amount of the enzyme that catalyzed the formation of 1 µmol of ONP per minute at 30 °C.

### 3.6. Effects of Temperature on the Activity and Stability of the Enzyme

The enzyme activity was measured at different temperatures ranging from 0 to 80 °C, at 10 °C intervals to determine the effect of temperature on the activity of the enzyme. The thermal stability of the enzyme was investigated by incubating the enzyme at temperatures ranging from 0 °C to 50 °C at 10 °C intervals for 1 h.

### 3.7. Effects of pH on the Activity and Stability of the Enzyme

The optimum catalytic pH of the recombinant enzyme was determined by measuring its activity in reaction mixtures with pH values ranging from 4.0 to 11.0. The pH stability of the enzyme was determined by measuring the residual activity after incubating the enzyme in the different buffers mentioned above at 4 °C for 1 h.

### 3.8. Effects of NaCl on the Activity and Stability of the Enzyme

The enzyme activity was assayed with 0.5–5 M NaCl added into the reaction mixture to determine the effects of NaCl on the catalytic activity of the enzyme. The NaCl tolerance of the enzyme was determined by measuring the residual activity after incubating the enzyme in buffers at various concentrations ranging from 0.5 M to 5 M of NaCl at 4 °C for 1 h.

### 3.9. Effects of Metal Ions and Chemicals on the Activity of the Enzyme

To determine the effects of metal ions and chemicals on the enzyme activity, 2 mM of NaCl, KCl, MgCl_2_, CaCl_2_, ZnCl_2_, MnCl_2_, NiCl_2_, FeCl_2_, FeCl_3_, SDS, EDTA·2Na, L-glutathione (oxidized) and 2% glycerol were added to the reaction mixture individually. No chemical was added to the control.

### 3.10. Effects of Organic Solvents on the Stability of the Enzyme

In order to determine the effects of organic solvents on enzyme stability, the residual activity was assayed after incubating the enzyme at different concentrations (volume fractions were 10%, 20%, 30%, 40%, and 50%) of DMSO, acetone, methanol, ethanol and acetonitrile at 4 °C for 1 h.

### 3.11. Substrate Specificity of the Enzyme

The substrate specificity of the enzyme was investigated using the standard β-galactosidase activity assay, except that ONPG was replaced by lactose, cellobiose, trehalose, maltose, arabinogalactan and pectic galactan, respectively.

### 3.12. Steady-State Kinetics

For steady-state kinetic studies, various concentrations of ONPG (0.1–10 mM), lactose (1–100 mM), Galβ-(1,3)-GlcNAc (1-40 mM) and Galβ-(1,4)-GlcNAc (1–50 mM) were used as substrates, respectively. When β-lactose was used as a substrate, the amount of glucose generated at different substrate concentrations was determined using the glucose oxidase method to calculate the kinetic parameters of rMaBGA [35]. When Galβ-(1,3)-GlcNAc and Galβ-(1,4)-GlcNAc were used as substrates, the reaction was terminated with 0.3 M NaOH and then neutralized with 0.3 M HCl [36]. Finally, the kinetic parameters were determined by measuring the amount of galactose produced at different substrate concentrations using an enzymatic coupling reaction technique including galactokinase, pyruvate carboxylase and formate dehydrogenase [37]. The kinetic constants of the enzyme were calculated using a nonlinear fitting of the Michaelis–Menten equation:

$$V=\frac{V_{max}[S]}{K_{m}+[S]}$$

where [*S*] is the substrate concentration, *K_m_* is the Michaelis constants for the substrate, *V* is the reaction velocity, and *V_max_* is the maximum reaction velocity. The turnover number *k_cat_* was calculated according to the following equation: *V_max_* = *k_cat_* [*E*], where [*E*] is the concentration of the enzyme.

### 3.13. Thermal Denaturation Kinetics

Thermodynamic parameters for irreversible thermal denaturation of the enzyme were determined by incubating enzymes at different temperatures. Aliquots were withdrawn at periodic intervals and cooled in an ice bath prior to the assay of the residual activity of the enzyme. The inactivation rate constants (*k_d_*) were calculated from a semi-logarithmic plot of residual activity versus time and the half-lives were calculated according to the following equation:

(1) $$t_{1/2}=0.693/k_{d}$$

The activation energy of denaturation (E_a_*) was calculated according to the Arrhenius plot:

(2) $$k_{d}=Ae^{-E_{a}^{*}/(RT)}$$

where *A* is the pre-exponential factor, *R* is the gas constant (8.314 J K^−1^ mol^−1^) and *T* is the absolute temperature.

The inactivation enthalpy (ΔH*), the free energy of inactivation (ΔG*) and the inactivation entropy (ΔS*) were calculated according to the following equations [38]:

(3) $$\Delta H^{*}=E_{a}^{*}-RT$$

(4) $${\Delta G}^{*}=-RT\ln(k_{d}h/k_{B}T)$$

(5) $$\Delta S^{*}=(\Delta H^{*}-\Delta G^{*})/T$$

where *h* is the Planck constant (6.63 × 10^−34^ J s) and *k_B_* is the Boltzmann constant (1.38 × 10^−23^ J K^−1^).

### 3.14. Analysis of Variance

Analysis of differences between groups was performed using IBM SPSS Statistics 22.0 software. For the analysis of differences between groups, one-way ANOVA (Analysis of Variance) was used and significance was analyzed according to the Least Significant Difference (LSD) method, with *p* ≤ 0.001 indicating a highly significant difference, 0.001 < *p* ≤ 0.01 indicating a highly significant difference, 0.01 < *p* ≤ 0.05 indicating a significant difference and *p* > 0.05 indicating a non-significant difference.

## 4. Conclusions

In this study, a putative β-galactosidase from *Marinomonas* sp. BSi20584 was successfully expressed in *E. coli* with a stable soluble form. The recombinant enzyme was purified to homogeneity and characterized extensively. The optimum temperature and pH of rMaBGA were determined as 50 °C and 7.0, respectively. The activity of rMaBGA was significantly enhanced by some divalent cations such as Zn^2+^, Mg^2+^ and Ni^2+^, but inhibited by EDTA, suggesting that some divalent cations might play important roles in the catalytic process of rMaBGA. Although the enzyme was derived from a cold-adapted strain, it still showed considerable stability against various physical and chemical elements. In addition, rMaBGA was able to hydrolyze both β-1,3 and β-1,4 glycosidic bond-linked substrates, which is a relatively rare occurrence in GH2 β-galactosidase. As an atypical GH2 β-galactosidase, two domains located at the C-terminal region of rMaBGA might have contributed to its β-1,3-galactosidase activity. On account of its fine features, this enzyme is a promising candidate for the industrial application of galactosidase.

## Figures and Tables

**Table 1:** Effects of metal ions and chemicals on the activity of rMaBGA *.

Metal Ions	Relative Activity (%)	Chemicals	Relative Activity (%)
Control	100 ^b^	Glycerol	134.846 ± 12.909 ^a^
Zn^2+^	135.857 ± 8.286 ^a^	L-glutathione (oxidized)	140.193 ± 3.047 ^a^
Mg^2+^	142.263 ± 0.423 ^a^	EDTA·2Na	40.141 ± 0.488 ^e^
Ni^2+^	121.218 ± 6.632 ^b^	SDS	45.161 ± 6.920 ^e^
K^+^	86.545 ± 3.926 ^cd^		
Na^+^	85.855 ± 2.771 ^cd^		
Fe^2+^	92.927 ± 4.478 ^bc^		
Fe^3+^	78.092 ± 1.936 ^d^		
Ca^2+^	98.620 ± 4.952 ^b^		

* Data represented as mean ± standard deviation of triplicate measurements. Data represented as mean ± standard deviation of triplicate measurements. Different letters indicate statistically significant differences (*p* ≤ 0.05).

**Table 2:** Substrate specificity of rMaBGA.

Natural Substrate	Substrate Activity (%)
Lactose	100
Trehalose	5.618
Maltose	1.053
Cellobiose	<1
Arabinogalactan	0
Pectic galactan	0

**Table 3:** Steady-state kinetics of rMaBGA ^a^.

Substrates	*K_m_* (mM)	*k_cat_* (s^−1^)	*k_cat_*/*K_m_* (s^−1^ mM^−1^)
ONPG	0.294 ± 0.044	13.160 ± 0.459	46.028 ± 8.450
β-lactose	5.265 ± 0.520	7.768 ± 0.212	1.494 ± 0.188
Galβ-(1,3)-GlcNAc	2.107 ± 0.484	0.025 ± 0.001	0.013 ± 0.004
Galβ-(1,4)-GlcNAc	7.023 ± 1.181	3.388 ± 0.156	0.500 ± 0.106

^a^ Data represented as mean ± standard deviation of triplicate measurements.

**Table 4:** Thermodynamics of irreversible thermal denaturation of rMaBGA.

Temperate (°C)	*k_d_* (h^−1^)	*t*_1/2_ (h)	ΔH* (kJmol^−1^)	ΔG* (kJmol^−1^)	ΔS* (Jmol^−1^K^−1^)
20	0.00153	452.94	118.19	87.55	104.52
30	0.0035	196.32	115.67	85.62	99.14
40	0.0116	59.94	113.07	82.77	96.73
50	0.189	3.67	110.38	81.24	90.18

## Data Availability

Not applicable.
